# Integrated Automatic Detection, Classification and Imaging of High Frequency Oscillations With Stereoelectroencephalography

**DOI:** 10.3389/fnins.2020.00546

**Published:** 2020-06-04

**Authors:** Baotian Zhao, Wenhan Hu, Chao Zhang, Xiu Wang, Yao Wang, Chang Liu, Jiajie Mo, Xiaoli Yang, Lin Sang, Yanshan Ma, Xiaoqiu Shao, Kai Zhang, Jianguo Zhang

**Affiliations:** ^1^Department of Neurosurgery, Beijing Tiantan Hospital, Capital Medical University, Beijing, China; ^2^Stereotactic and Functional Neurosurgery Laboratory, Beijing Neurosurgical Institute, Capital Medical University, Beijing, China; ^3^Beijing Key Laboratory of Neurostimulation, Beijing, China; ^4^China National Clinical Research Center for Neurological Diseases, Beijing, China; ^5^Department of Neurosurgery, Beijing Fengtai Hospital, Beijing, China; ^6^Department of Neurology, Beijing Tiantan Hospital, Capital Medical University, Beijing, China

**Keywords:** high frequency oscillations, epileptogenic zone, epilepsy surgery, stereoelectroencephalography, convolutional neural network

## Abstract

**Objective:**

During presurgical evaluation for focal epilepsy patients, the evidence supporting the use of high frequency oscillations (HFOs) for delineating the epileptogenic zone (EZ) increased over the past decade. This study aims to develop and validate an integrated automatic detection, classification and imaging pipeline of HFOs with stereoelectroencephalography (SEEG) to narrow the gap between HFOs quantitative analysis and clinical application.

**Methods:**

The proposed pipeline includes stages of channel inclusion, candidate HFOs detection and automatic labeling with four trained convolutional neural network (CNN) classifiers and HFOs sorting based on occurrence rate and imaging. We first evaluated the initial detector using an open simulated dataset. After that, we validated our full algorithm in a 20-patient cohort against three assumptions based on previous studies. Classified HFOs results were compared with seizure onset zone (SOZ) channels for their concordance. The receiver operating characteristic (ROC) curve and the corresponding area under the curve (AUC) were calculated representing the prediction ability of the labeled HFOs outputs for SOZ.

**Results:**

The initial detector demonstrated satisfactory performance on the simulated dataset. The four CNN classifiers converged quickly during training, and the accuracies on the validation dataset were above 95%. The localization value of HFOs was significantly improved by HFOs classification. The AUC values of the 20 testing patients increased after HFO classification, indicating a satisfactory prediction value of the proposed algorithm for EZ identification.

**Conclusion:**

Our detector can provide robust HFOs analysis results revealing EZ at the individual level, which may ultimately push forward the transitioning of HFOs analysis into a meaningful part of the presurgical evaluation and surgical planning.

## Introduction

Although a majority of seizures can be well controlled by antiepileptic drugs, approximately 30% of patients suffer from uncontrolled seizures despite pharmacotherapy, who are potential candidates for presurgical evaluation and subsequent surgery interventions (Devinsky, 1999; Jobst and Cascino, 2015). Accurate localization and safe removal of the EZ are major prognostic factors for good surgical outcomes (Vakharia et al., 2018). Intracranial EEG recordings are often used to identify the epileptogenic regions, especially in MRI negative cases, for their capability of direct recording of epileptogenic discharges from brain parenchyma with high temporal and spatial accuracy, and they have been considered an electrophysiological gold standard for delineating SOZ, which defines EZ to a large extent (Rosenow and Luders, 2001). SEEG using depth electrodes has been more widely adopted in recent years because it is superior for recording deep brain structures and less invasive compared with the subdural grid electrode approach (Nagahama et al., 2018; Zijlmans et al., 2019). Currently, epileptologists mainly focus on ictal SEEG to reveal SOZ; however, interictal HFOs have increased in popularity as a promising biomarker for the EZ over the past decade (Bragin et al., 1999; Jacobs et al., 2008).

It has been well illustrated and replicated that the rates of HFOs were higher within the SOZ than outside (Worrell et al., 2004; Urrestarazu et al., 2007; Jacobs et al., 2008). From a surgical perspective, several studies have shown that tailored resection of HFOs regions predicts better surgical outcomes and that residual HFOs are prognostic markers for seizure recurrence (Wu et al., 2010; van Klink et al., 2014; van ’t Klooster et al., 2015). In addition, a recent meta-analysis also indicated a significant relationship between the removal of tissue with high HFOs rates and surgical outcomes (Holler et al., 2015).

HFOs are characterized as transient small and fast oscillating phenomenon, which typically last 6–30 ms with varied morphometry (Zijlmans et al., 2017). They can also sometimes be mislabeled due to impulse-like artifacts contamination and improper filtering (Benar et al., 2010). Therefore, it is well acknowledged that manual detection of HFOs can be extremely laborious, time-consuming and prone to subjective bias (Lopez-Cuevas et al., 2013; Spring et al., 2018). Under this background, a variety of automated detection algorithms have been developed, which were implemented to help limit the manpower required for HFO analysis significantly and to avoid the bias induced by human raters (Thomschewski et al., 2019). However, most HFO detection algorithms have been conducted through simply thresholding instantaneous frequency traces, which might be vulnerable to the influence of artifacts and the irregular morphometry of HFOs (Chaibi et al., 2013).

During clinical application of HFOs, not only is the detection accuracy important, but the classification of various events is also crucial. HFOs can be categorized into ripples (Rs, 80–250 Hz) and fast ripple (FRs, 250–500 Hz) according to their frequency range (Jacobs et al., 2012). FRs are reported to be more focal and closely linked to epileptogenicity than Rs (Engel et al., 2009; Akiyama et al., 2011). Evidence indicates that HFOs cooccurring with a spike were more closely related to the SOZ (Wang et al., 2013). In addition, artifacts due to muscle activity or bad connections result in significantly more FP findings. Therefore, a two-stage detection and classification framework was proposed and has achieved high sensitivity in recent years while maintaining high specificity by identifying different types of events at the second stage (Zijlmans et al., 2017). Under such a framework, some detectors have been developed as semiautomatic, requiring visual validation (Navarrete et al., 2016), while others have implemented fully automated postprocessing steps such as feature extraction and clustering for the classification problem (Gliske et al., 2016; Liu et al., 2016a).

For discriminating false HFOs or any other events, it has been suggested that time-frequency (TF) representation of HFOs is highly beneficial in distinguishing events of different types (Benar et al., 2010). Therefore, the event discrimination task can be categorized as a two-dimensional time-frequency image classification problem. Convolutional neural network (CNN)-based models are promising techniques that have been applied successfully for classifying images, and they have gained momentum in recent years for their advantageous performance over traditional models (Krizhevsky et al., 2012). Therefore, we hypothesized that CNN image classifiers can also be used for the HFO classification problem with good efficiency.

Although several HFOs detection algorithms have been published, the general validation and clinical value of these approaches are less well addressed in the clinical application aspect (Zijlmans et al., 2017). For ease of clinical use, the following merits should be considered for any efficient HFOs analysis tools. First, it should maintain high sensitivity and specificity when detecting HFOs. Second, the requirement for user input parameters and intervention should be minimized to save labor and reduce bias. Third, the algorithm should have robust classification ability to identify artifacts, subtypes of HFOs and HFOs cooccurring with other interictal epileptiform discharges. Fourth, because the final goal for any HFO detector is to locate the EZ through the distribution of HFOs, the clinical value of the algorithm should be evaluated and validated. Finally, the results should be properly projected on anatomical structures to facilitate surgical planning. Motivated by the clinical need for efficient and reliable HFOs analysis tool, the proposed algorithm attempted to cover the abovementioned properties and provided a comprehensive solution for HFO analysis. The automatic procedures mimic typical manual analysis, which includes channel selection (excluding channels outside the brain, with clear continuous artifacts or located in white matter), HFOs detection, classification and anatomical projection.

Overall, our detection algorithm adopted the two-stage framework containing an initial detector and a CNN-based classifier. We first validated the initial detector through a labeled simulated dataset built by Roehri et al. (2017). To further validate the clinical values of this pipeline, we retrospectively applied the method to intracranial EEG data recorded from 20 patients who underwent resection surgery with good surgical outcomes but with different pathological substrates. Taking advantage of the labeled events after classification, we further validated the results in a real dataset against some established findings in the literature while evaluating its performance for predicting SOZ.

## Materials and Methods

### Stages of the Proposed Pipeline

#### Channel Selection

Preimplantation 1 mm isotropic T1 weighted MRI images and the coordinates for each depth electrode contact in individual space, which were determined by coregistering the post implantation computerized tomography image with the preimplantation MRI, and raw SEEG data were collected. First, brain extraction was performed by ROBEX (Iglesias et al., 2011) to make a binary brain mask to identify outside brain contacts. Raw SEEG data were first subjected to a notch filter for line noise of 50 Hz and its harmonic up to 450 Hz using a 3-order Butterworth filter and then were re-referenced offline in a bipolar manner. Any bipolar SEEG channel containing outside brain contact was excluded from further analysis to avoid artifacts. Then, a binary gray matter mask was produced by the SPM12^1^ unified segmentation procedure with a threshold of 0.4. A 3 × 3 × 3 voxel cube centered on the middle point of the 2 adjacent contacts was modeled to identify whether the bipolar channel was localized in the white matter. The bipolar channel was labeled as out of gray matter if the 3 × 3 × 3 voxel cube contained fewer than 9 gray matter voxels. The bipolar channel was labeled as low amplitude if the corresponding root mean square (RMS) was below 35% of all channels, formerly excluding those labeled as out brain. Only channels labeled as out of gray matter and low amplitude were excluded for further analysis. Channels with extreme high-amplitude noise (voltage > 1000 uV) lasting for more than 1 s were also excluded from the analysis. This stage ensured that most artifact contaminated channels as well as low-amplitude channels in white matter were screened out from further analysis.

#### Candidate HFO (cHFO) Detection and Automatic Labeling

After the channel selection described above, the baseline-corrected SEEG segments were first filtered using a 64-order zero-phase forward and reverse bandpass FIR filter in the 80–500 Hz range. Then, the rectified filtered signal envelope was determined using spline interpolation over local maxima. The SDs of the bandpass filtered signal in each 100 ms epoch was calculated for the whole time series with a step length of 100 ms, generating a distribution of SDs for each channel. An amplitude threshold was set to five times the median of SDs (Staba et al., 2002; Liu et al., 2016b). A cHFO was defined if the envelope surpassed the amplitude threshold and lasted more than 6 ms. The maximum envelope peaks of putative HFOs separated by fewer than 20 ms were considered as one event. According to the 1/f law, the amplitude of FRs was lower than Rs, so we repeated the aforementioned procedures with 250–500 Hz bandpass signals and detected extra events to form the final cHFOs for further classification.

The cHFOs were extracted and epoched in 600 ms windows centered on local envelope peaks, and the Morlet wavelet (central frequency equals 1 Hz, and the full-width at half-maximum equals 3 s) TF transform (1–500 Hz with step length equals 1 Hz) was applied to generate the scalograms (Pantazis et al., 2005). To decrease the impact of the 1/f spectrum on the scalogram, we bandpass filtered the raw data with a first-order Butterworth 8–490 Hz while preserving most of the low-frequency features, especially spikes from the raw traces, before the TF transform. The raw power was log-transformed and smoothed for better visualization as TF maps. We extracted the central 200 ms window TF maps as the final training and classification dataset to avoid edge effects during the TF transform. Considering the typical duration of HFOs (6–30 ms) and interictal epileptiform discharges such as spike (30–70 ms) and sharp wave (70–200 ms) (Aanestad et al., 2020), we think that the 200 ms window will sufficiently cover the whole spectrum characteristics of the detected events.

After some trials on a small training and validation subset, ResNet101 was finally chosen since it yielded the best results with the least overfitting out of AlexNet, GoogLeNet, VGG-16 (Alom et al., 2018). Four pretrained convolutional neural network Resnet101 were trained through transfer learning as binary classifiers with purposes of labeling artifacts, spikes, Rs and FRs in sequence order. Details of Resnet101 can be found in He et al. (2016). Transfer learning was implemented by replacing the last 3 layers of the pretrained ResNet101 to a new fully connected layer, softmax layer and class-output layer in sequence. The whole TF image datasets included 29,744 artifacts events against 68,988 non-artifacts events, 30,387 spike events against 23,452 non-spike events, 38,447 R events against 22,152 non-R events, and 26,454 FR events against 27,695 non-FR events. The image dataset was composed of a mix of real signals and simulated data described below except that all the artifact TF images were generated from real data. The main source of artifacts was electromyography and sharp transients. The real training materials were extracted from 12 consecutively selected drug-refractory epilepsy patients in Beijing Tiantan hospital from June 2018 to July 2019 (detailed clinical information is provided in Supplementary Table 1). Two experienced reviewers worked independently to label the events, and only those events with the same conclusion from both reviewers were included in the dataset. We randomly divided the data into training and validation datasets, using 80% of the images for training and 20% for validation. The training process used stochastic gradient descent with a momentum of 0.9. We used cross entropy as a loss function. The minibatch size was set to 32, max epochs to 3 and initial learning rate to 0.0001. To further validate the robustness of the training process, we repeatedly trained the network by using 70% and 90% of the images for training and 30% and 10% for validation. The training processes were stopped manually when the loss/accuracy plateau was reached to avoid overfitting, and the accuracies on the test dataset were recorded. Theoretically, the four consecutive classifiers would output 9 reasonable categories of labels, which were artifacts, spike, R, FR, spike + R, spike + FR, spike + R + FR, R + FR and other (defined by four negative predictions and treated as artifacts). After event labeling, cHFO not concurrent with artifacts were labeled quality HFO (qHFO).

#### HFOs Occurrence Rate Sorting and Imaging

**FIGURE 1:**
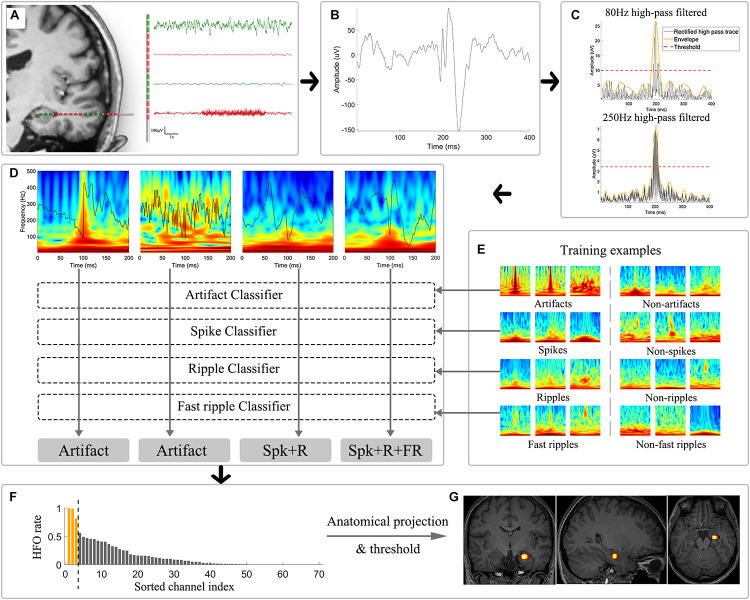
Schematic illustration of the automatic analytical strategies. **(A)** Channel selection was performed to exclude electrodes located in white matter, showing low-amplitude fluctuation and located outside brain (plotted in red). **(B)** Example of an unfiltered bipolar signal in a 400 ms window. **(C)** Signals were independently subjected to 80 and 250 Hz high-pass filters and were then rectified (black trace) for envelope extraction (yellow trace). Thresholds (red trace) were calculated based on the envelopes. Candidate HFOs were extracted by identifying envelopes surpassing the corresponding threshold of each channel. **(D)** Morlet wavelet transform was applied to convert the epoched candidate HFO time series to time-frequency domain images, which were further used as input for the CNN classifiers. **(E)** Example training and validation dataset used for the four binary CNN classifier training. **(F)** HFOs sorting based on the occurrence rate. The yellow bar suggested the application of thresholding in HFOs occurrence rate that would be overlaid on the anatomical image. **(G)** The results were then projected to the anatomical structures and shown as a heatmap illustrating the distribution of high-occurrence HFOs. CNN: convolutional neural network; HFOs: high frequency oscillations; Spk: spike; R: ripple; FR: fast ripple.

The occurrence rate was normalized to the highest occurrence rate channel. Normalized value assignment was performed in every voxel included in the volume defined by a 9 × 9 × 9 mm cube model centered on each electrode contact (David et al., 2011). The results were smoothed through a [4 4 4] Gaussian kernel, which was overlayed on volumes. The pipeline was developed in MATLAB 2018a (The MathWorks, Inc., Natick, MA, United States) and illustrated in Figure 1. Codes of the algorithms described in this paper, including the trained neural network, are open-source and openly available^2^.

### Evaluation of the Initial Detector Using Simulated Data

The simulated dataset provided an ideal testing environment featured by its artifact-free signals and well-controlled signal-to-noise ratios (SNRs) of HFOs. We adopted the HFOs dataset simulated by Roehri et al. (2017) since it was already tested against four state-of-the-art openly available detectors, namely the Short Time Energy, the Short Line Length, the Hilbert and the MNI detector from RIPPLELAB Toolbox (Navarrete et al., 2016). Thus, the benchmarks of our detector can be directly compared with those open detectors. Specifically, the dataset included 960 channels lasting for 2 min with a sampling rate of 2,048 Hz. Each channel contains 42 inserted events of 7 different types: 1. spike; 2. spike cooccurring with an R; 3. spike cooccurring with an FR; 4. spike cooccurring with an R and an FR; 5. R; 6. FR; 7. R cooccurring with an FR. Following the suggestions by the authors of the dataset, we defined a 100 ms time window centered on each simulated HFO as the CI. CIs containing detections were considered true positives (TP), those without detections were defined as FN, and detections falling outside CIs were labeled as FPs. We used the precision (Prec_detection_) and sensitivity (Sens_detection_) criteria as well as the F1-score, which combines precision and sensitivity, to characterize the detection performance over the SNRs. The precision and F1-score were defined as 1 and 0, respectively, when no event was detected (i.e. TP + FP = 0) in 0 dB channels.

$$S\,e\,n\,s_{d\,e\,t\,e\,c\,t\,i\,o\,n}=\frac{\text{TP}}{\text{TP}+\text{FN}}$$

$$P\,r\,e\,c_{d\,e\,t\,e\,c\,t\,i\,o\,n}=\frac{\text{TP}}{\text{TP}+\text{FP}}$$

$$\text{F}\,1-\text{score}=\frac{2\,\text{TP}}{2\,\text{TP}+\text{FN}+\text{FP}}$$

### Testing Cohort and SEEG Recordings

Patients from Beijing Tiantan Hospital and Beijing Fengtai Hospital following the criteria between October 2015 and October 2017, were included in this analysis retrospectively: (1) unifocal epilepsy confirmed by analysis of SEEG seizure onsets; (2) surgical resection after the SEEG; (3) the sampling rate of EEG amplifier >2000 Hz; and (4) postoperative Engel I with at least a 24-month follow-up. Only Engel I patients were included so that we could assume that the EZ was correctly identified. This study was approved by the Ethics Board of the Beijing Tiantan Hospital, Capital Medical University. Informed consent was given by patients or their legal guardian/next of kin about the use of data for research purposes.

The SEEG recording was carried out as part of the clinical routine of the included patients. Intracerebral multiple contact depth electrodes (Huake-Hengsheng Medical Technology, Beijing, China; 8–16 contacts, length: 2 mm, diameter: 0.8 mm, 1.5 mm apart) were placed using a CRW frame-based system (Integra Radionics, Burlington, MA, United States) to record intracranial EEG data. The strategy for electrode placement, independent from the present study, was based on noninvasive information providing clinical hypotheses about the localization of the EZ. Twenty-four hours after electrode implantation, electrophysiological signals were recorded on a video EEG system (Nihon-Kohden, Tokyo, Japan). Long-term SEEG monitoring was carried out to record at least two habitual seizures. The built-in antialiasing hardware bandpass filter of the amplifier was set to 0.08–600 Hz for a 2000 Hz sampling rate. Typical monitoring sessions lasted from 7 days up to 1 month. We randomly selected one 5–10 min clip from each patient without selection of electrode contacts, patient’s state (awake/sleep), or quality of recordings.

### Concordance of HFOs Results and SOZ

In clinical situations, all the depth electrodes were implanted according to the consensus reached during the phase I evaluation. The medical history, scalp EEG, ictal semiology, structure MRI and fluorodeoxyglucose-positron emission tomography were reviewed and discussed. In all patients presented here, SOZ was independently visually identified by two senior epileptologists (Xiao-qiu Shao and Wen-han Hu) by reviewing and labeling the channels with the earliest ictal discharge during recorded seizures. The SOZ was taken as the gold standard guiding surgical planning of resection in individual bases.

To evaluate the SOZ prediction ability of this algorithm, sensitivity (Sen_SOZ_), specificity (Spec_SOZ_) and ROC curve with AUC were calculated and served as quantitative parameters. Sensitivity and specificity were defined as (Burnos et al., 2014):

$${\text{Sens}}_{\text{SOZ}}=\frac{{\text{CH}}_{\text{HFO}}\,\mathrm{in}\,\mathrm{SOZ}}{{\text{CH}}_{\text{HFO}}\,\mathrm{in}\,\mathrm{SOZ}+{\text{CH}}_{\text{Non}-\text{HFO}}\,\mathrm{in}\,\mathrm{SOZ}}$$

$${\text{Spec}}_{\text{SOZ}}=\frac{{\text{CH}}_{\text{Non}-\text{HFO}}\,\mathrm{not}\,\mathrm{in}\,\mathrm{SOZ}}{{\text{CH}}_{\text{Non}-\text{HFO}}\,\mathrm{not}\,\mathrm{in}\,\mathrm{SOZ}+{\text{CH}}_{\text{HFO}}\,\mathrm{not}\,\mathrm{in}\,\mathrm{SOZ}}$$

We manually set threshold to the first N channels with the highest HFOs rate to be CH_HFO_ and the rest to be CH_Non–HFO_. The ROC curve was obtained by plotting the Sen_SOZ_ as a function of the (1 - Spec_SOZ_) at each cutoff N, which was increased from one to the number of total contacts in each patient with a step length of one. The corresponding AUC of each patient was calculated using the trapezoid method.

Although the accuracy of the four classifiers was evaluated in the validation set, to further test their efficiency and generalization ability in the clinical environment, we next conducted an additional analysis with hypotheses in relation to the automatic classifier that (1) the localization value of HFOs could be enhanced by eliminating artifacts; (2) HFOs co-occurring with spike have better predictive value for SOZ than those without. (3) HFOs with FR are more closely related to SOZ than those without.

## Results

### Performance of the Initial Detector on the Simulated Data Set

**FIGURE 2:**
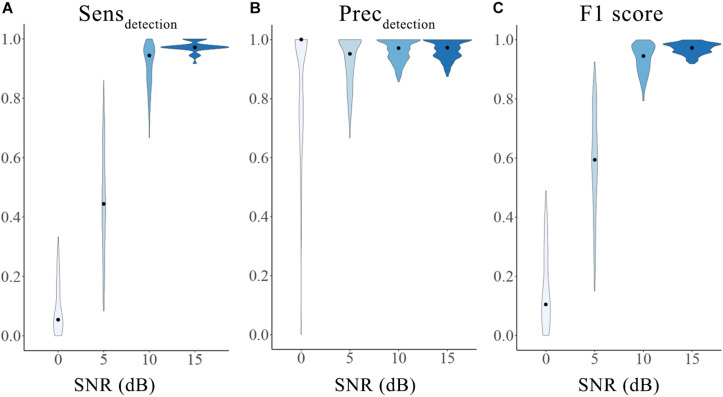
Performance of the initial detector tested on an open simulation dataset. The three metrics were calculated as sensitivity **(A)**, precision **(B)** and F1 score **(C)** for different SNRs. The violin plots show the range (minimum to maximum) and distribution of the data. The black dots represent the median values. Different color indicates different SNRs groups. SNRs: signal-to-noise ratios; Sens_detection_: sensitivity of the initial detection; Prec_detection_: precision of the initial detection.

An accurate initial detector lays a solid foundation for the integrated HFOs detection and classification framework. Taking advantage of the well-established simulated HFOs dataset, we were able to evaluate the overall performance of the initial detector using the 3 metrics described in the “Materials and Method” section. As expected, the performance of the automatic detector increased with SNRs. The median and range of sensitivity, precision and F1-score for different SNRs were 0 dB [5.56%, 0–33.3%], 5 dB [44.44%, 8.33–86.11%], 10 dB [94.44%, 66.67–100%], 15 dB [97.22%, 91.67–100%]; 0 dB [100%, 0–100%], 5 dB [95.12%, 66.67–100%], 10 dB[97.14%, 85.71–100%, 15 dB [97.22%, 87.50–100%]; 0 dB [0%, 0–48.98%], 5 dB [59.45%, 15.00–92.54%], 10 dB [94.44%, 79.37–100%], 15 dB [97.22%, 91.89–100%], respectively. The results suggest satisfactory and stable accuracy of the initial detector, especially when the SNR was high (10 dB and 15 dB). The distributions of the sensitivity, precision and F1-score of different SNRs are shown in Figure 2. Generally speaking, our initial detector outperformed the Short Time Energy, the Short Line Length, the Hilbert and the MNI detector from RIPPLELAB Toolbox and was comparable with Delphos detector (Roehri et al., 2017).

### Classification Accuracy of the Trained CNN Classifiers

Four Resnet101 networks, namely, artifacts, spike, R and FR classifiers, were trained. The loss function generally converged quickly, and the loss/accuracy plateaus were reached after 2–3 epochs. Specifically, after the last iteration, the prediction accuracies in the validation set for artifacts, spike, R and FR classifiers of different training/validation split ratios were summarized in Table 1. It is worth noting that we divided the whole dataset into training and validation groups rather than training, validating and testing groups since we adopted a pretrained ResNet-101 network, which did not require intensive structure modification or hyperparameter tuning during training and validation processes. The training process showed robustness across different split ratio and no overfitting problem occurred.

**TABLE 1:** Training results of different split ratios for ResNet101 classifiers.

**Classifiers**	**Accuracies from different training/validation split ratio**		
	**70%**	**80%**	**90%**
Artifacts	98.98%	99.03%	99.18%
Spike	97.93%	98.11%	98.12%
Ripple	95.65%	95.97%	95.99%
Fast ripple	96.46%	96.55%	96.71%

### Demographics and HFOs Detection and Classification Results of the Testing Cohort

In general, 20 consecutive patients (4 female) met the inclusion criteria and were included as the testing cohort. Their pathological results varied, including hippocampal sclerosis, focal cortical dysplasia and tuberous sclerosis complex (TSC). Detailed demographics and clinical information are provided in Supplementary Table 2. In total, 2,048 channels (mean 102.4, range 57–187) were fed into the pipeline, and the mean duration of interictal clips for each patient was 9.2 min (range 4.2–11.7 min) for each subject. Based on the channel inclusion criteria automatically implemented by the algorithm, 142 (6.9%), 254 (12.4%) and 22 (1.1%) channels were identified as outside the brain, inside white matter and with extreme amplitude values, respectively, which were excluded from further analysis. A total of 125,567 cHFOs were detected during the first stage. The detailed detection and classification outputs are provided in Table 2. Representing example figures of the labeled artifact, spike + R, spike + R + FR can be found in Figure 1D.

**TABLE 2:** Detection and classification results and the AUC values regarding SOZ of 20 testing patients.

**Patient**	**Clip duration (min)**	**Channel selection**				**Event classification**									**AUC values**		
		**Total**	**WM**	**OB**	**EV**	**Total**	**Art./others**	**Spk**	**R**	**FR**	**Spk + R**	**Spk + FR**	**Spk + R + FR**	**R + FR**	**cHFOs**	**qHFOs**	**qHFOs w/FR**
01	9.7	121	13	9	0	15865	743	196	1970	21	9081	44	3770	40	0.764	0.764	0.757
02	11.7	120	17	6	7	10403	3655	32	1874	107	1633	28	2276	798	0.956	0.980	0.962
03	5.0	81	13	2	1	2482	71	26	469	5	694	9	1154	54	0.946	0.946	0.949
04	7.0	115	22	2	2	981	92	29	63	11	278	68	432	8	0.996	1.000	0.992
05	9.6	136	16	12	2	643	211	3	137	6	131	3	142	10	0.998	0.998	1.000
06	10.8	58	2	7	0	10427	1267	30	392	288	2568	209	5424	249	0.855	0.893	0.889
07	4.5	100	5	10	0	4435	781	52	476	161	777	210	1815	163	1.000	1.000	1.000
08	4.2	70	5	2	2	2781	495	47	284	66	624	77	1130	58	0.920	0.946	0.939
09	11.3	111	8	11	0	6338	2580	37	330	72	1632	30	1606	51	0.989	0.991	0.993
10	10.1	76	8	9	0	2755	682	52	600	45	530	81	693	72	0.973	0.997	0.995
11	9.7	104	17	7	0	4131	492	12	2771	14	417	5	364	56	0.633	0.658	0.840
12	9.7	100	19	7	0	1757	110	19	663	2	462	11	462	28	1.000	1.000	0.945
13	10.8	60	2	4	1	5074	912	53	2509	58	1283	5	142	112	0.980	0.987	0.967
14	6.7	187	27	10	2	9085	832	68	2273	143	2692	218	2658	201	0.985	0.986	0.926
15	10.2	126	17	8	2	11256	1367	136	1095	96	3291	187	4905	179	0.990	0.989	0.993
16	11.1	105	15	12	1	10709	323	108	1366	148	5344	59	3207	154	0.791	0.782	0.840
17	11.2	128	8	7	0	10717	222	58	2529	11	5153	17	2612	115	1.000	0.998	0.981
18	11.1	57	12	4	0	9657	442	230	598	21	3898	108	4296	64	0.992	1.000	0.992
19	11.0	118	11	8	1	4215	1677	19	583	18	1161	7	717	33	0.775	0.839	0.839
20	9.5	75	17	5	1	1856	369	4	80	5	289	7	1089	13	0.978	1.000	1.000
Total	184.8	2048	254	142	22	125567	17323	1211	21062	1298	41938	1383	38894	2458	/	/	/

*WM: channels identified in white matter; OB: channels identified outside brain; EV: channels including extreme amplitude values; Art.: artifacts; Spk: Spike; R: ripple; FR: fast ripple; cHFOs: candidate high frequency oscillations; qHFOs: quality high frequency oscillations; AUC: area under the curve.*

### Concordance Between Labeled HFOs and SOZ

To a large extent, the ultimate goal for any HFO detector is to provide interpretable results revealing EZ; therefore, we decided to compare the concordance between labeled HFOs and SOZ with the dual purpose of validating the classification results against the assumptions based on previous studies and further evaluating the diagnostic ability of this detector for identifying the EZ in a real dataset.

SOZ channels were visually marked by capturing the earliest ictal epileptic discharges identified by neurologists in 20 patients based on at least one ictal SEEG. In total, 127 channels (mean 6.4, range 1–14) were labeled as SOZ, and detailed SOZ channel information can be found in Supplementary Table 2. Instead of thresholding and simply comparing the overlap between HFO channels and SOZ channels, we chose to calculate the ROC curves and corresponding AUC value for each subject, representing the ability for SOZ localization of this algorithm.

**FIGURE 3:**
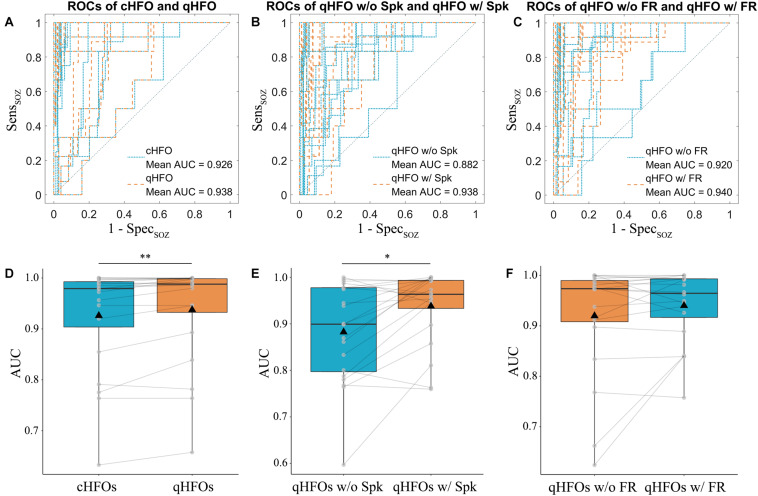
ROC curves and AUC values of different event types in the testing cohort. ROC curves were plotted for each patient comparing cHFOs and qHFOs, **(A)** qHFOs with and without spike **(B)** and qHFOs with and without FR **(C)**. The corresponding AUC values were calculated with the trapezoid method. Significant differences were found between cHFOs and qHFOs (*p* = 0.0043, Wilcoxon signed-rank test) **(D)**, qHFOs with and without spike (*p* = 0.0111, Wilcoxon signed-rank test) **(E)**, but not qHFOs with and without FR (*p* = 0.4209, Wilcoxon signed-rank test) **(F)**. *p < 0.05, **p < 0.01. ROC: receiver operating characteristic; AUC: area under the curve; HFOs: high frequency oscillations; cHFO: candidate HFOs; qHFOs: quality HFOs; FR: fast ripple; w/: with; w/o: without.

AUC values for different representative event types were compared at the group level. The median (interquartile range) of AUC values for cHFOs and qHFOs were 0.979 (0.106) and 0.988 (0.080) (*p* = 0.0043, Wilcoxon signed-rank test), suggesting the effect of artifact removal on improving the localization value. After excluding all artifact events, qHFOs with spike versus qHFOs without spike exhibited significant differences (*p* = 0.0111, Wilcoxon signed-rank test) and the corresponding median (interquartile range) of AUC values were 0.964 (0.073) and 0.899 (0.184), indicating a better prediction value of qHFOs with spike than those without for identifying the EZ. Likewise, we further compared the localization value of qHFO with FR and without, and the median (interquartile range) of AUC values were 0.964 (0.086) and 0.974 (0.087), respectively. The comparison result was statistically insignificant (*p* = 0.4209, Wilcoxon signed-rank test). See Figure 3 for the statistic comparisons above. We speculate that this phenomenon might partially be attributed to the ceiling effect since the AUC values clustered close to 1 in those 2 groups as well as the theory that FR were generated by more restricted regions compared to ripples (Bragin et al., 2002; Gonzalez Otarula et al., 2019). Although the difference was not significant, separating qHFOs with FR largely increased the two lowermost AUC values from 0.624 and 0.662 to 0.840 and 0.840, resulting in higher mean AUC values for qHFOs with FR.

**FIGURE 4:**
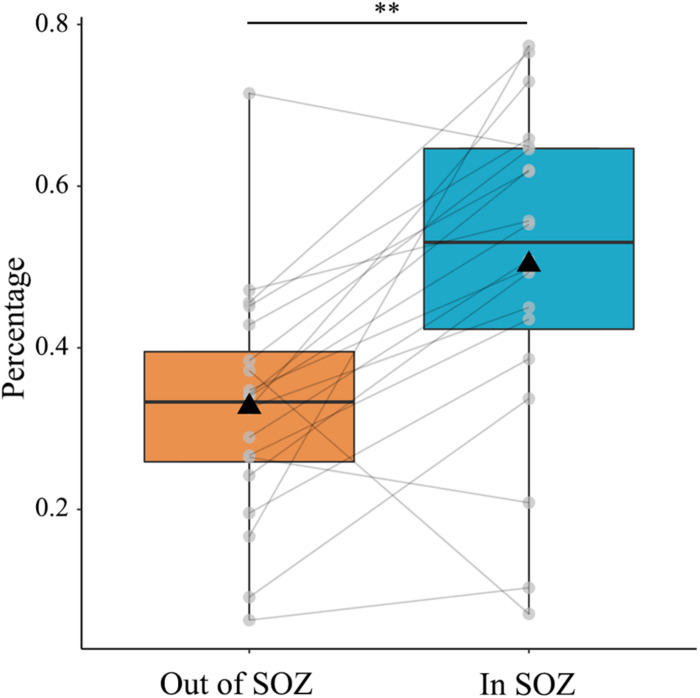
Percentage of qHFOs with FR inside and out of SOZ. The percentage of qHFOs with FR was significantly higher inside SOZ than outside (*p* = 0.0005, paired *t*-test). HFOs: high-frequency oscillations; FR: fast ripple; SOZ: seizure onset zone; qHFOs: quality HFOs.

**FIGURE 5:**
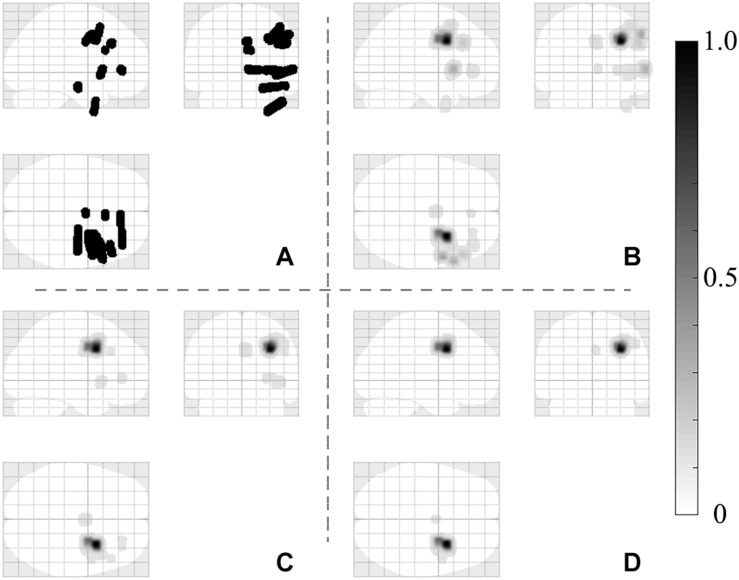
HFOs imaging plotted on a glass brain in Montreal Neurological Institute space showing **(A)** the automatic labeled gray matter depth electrode coverage; **(B)** the spatial distribution of cHFOs; **(C)** the distribution of qHFOs and the spatial distribution of qHFOs with FR **(D)**. It can be seen from the figures that with classification, the result was more specific and localized, indicating SOZ. No thresholding was used in the above figures. The intensity bar represents the min-max normalized HFOs occurrence rates. HFOs: high frequency oscillations; cHFO: candidate HFOs; qHFO: quality HFOs; FR: fast ripple; SOZ: seizure onset zone.

Next, we sought to compare the proportion of qHFOs with FR outside and inside SOZ. We hypothesized that the proportion of qHFOs with FR was higher inside SOZ than outside qHFO channels based on previous studies. The results illustrated in Figure 4 confirmed our hypothesis (mean ± SD: 0.503 ± 0.202 versus 0.327 ± 0.146, *p* = 0.0005, paired *t*-test). The above significant differences still existed after Bonferroni correction for multiple comparisons. To present an intuitive imaging illustration of the efficacy of classification, we plotted the density map of the HFOs rate on the glass brain in Montreal Neurological Institute space without thresholding as Figure 5.

## Discussion

Even HFOs have been recognized as a promising biomarker for identifying the EZ in recent years, analysis of HFOs is still challenging, mainly due to their usual low signal-to-noise ratio, their heterogeneous patterns and their association with other epileptic activity (Thomschewski et al., 2019). Automatic detection of HFOs has a considerable advantage over visual marking in terms of efficiency. Different algorithms, such as the short-time energy detector (Staba et al., 2002), the short line length detector (Gardner et al., 2007), the Hilbert detector (Crepon et al., 2010), and the Montreal Neurological Institute detector (Zelmann et al., 2012), have been published and are publicly available through the RIPPLELAB Toolbox (Navarrete et al., 2016); however, many publicly available detectors face challenges such as artifact contamination and the lack of robustness across different situations (i.e. low inter-method reproducibility), which impede their clinical implementation (Frauscher et al., 2017). To narrow the gap between HFOs analysis and clinical EZ localization, we designed an integrated pipeline imitating the current workflow of HFOs analysis. We have also systematically validated the performance of our detector in simulated datasets and real datasets. Features of the proposed pipeline include (1) channel selection based on anatomical localization and RMS to exclude flat channels and minimize the influence of artifacts; (2) automatic detection of cHFO through filtering and amplitude thresholds followed by labeling detected events with tags of spike, R and FR using a deep convolutional neural network in a supervised manner; and (3) visualization of high-rate HFOs channel distribution projected on brain structures.

Many automatic and semiautomatic algorithms have been developed and have shown promising results. However, apart from those flourishing studies, researchers should still be cautious about the sensitivity and specificity of their SOZ localization values. Part of the reason could be attributed to the lack of a gold standard for identifying HFOs. A working definition is that oscillatory activities in a frequency band from 80 to 500 Hz clearly stand out from the baseline signal and persist for at least four oscillation cycles. This definition may provide practical guidelines for the manual identification of HFOs; however, it lacks specific parameters needed for designing automatic detectors (Roehri et al., 2017). In addition, the literature constantly indicates that manual labeling can act as the gold standard (Amiri et al., 2016; Zuo et al., 2019), but manual labeling has been questioned in terms of subjective bias and interrater reliability. Here, as the base of our initial detector, we adopted a traditional yet feasible definition by Anatol Bragin et al. that successive RMS values with amplitudes of 5 SDs above the mean amplitude of the RMS signal longer than 6 ms in duration (Staba et al., 2002) with subtle modification as described in the “Materials and Method” section. As was discussed in the paper by Roehri et al. (2017), it was challenging to directly compare the performance between detectors because of the lack of gold standard, therefore, they proposed a benchmark framework and an openly available simulated dataset to ease the problem. The reason we choose the simulated dataset to validate our initial detector is that there are benchmarks of four openly available detectors based on this dataset and we can directly compare our detector with others. To evaluate the initial detector in a systematic manner, we tested our initial detector using the simulated dataset, and the performance was satisfactory, especially when the SNRs were high and robust compared with other four state-of-the-art openly available detectors from RIPPLELAB Toolbox (Navarrete et al., 2016).

Even though the performance was inspiring in the simulated dataset, we should keep in mind that the real dataset might be complicated, containing artifacts, physiology HFOs and pathological HFOs with various patterns (Engel et al., 2009; Kovach et al., 2011; Cimbalnik et al., 2018). Therefore, the clinical translation of HFOs as a biomarker of EZ has been largely limited by the ability to reliably detect and accurately classify HFOs (Khadjevand et al., 2017). With the purpose of improving the specificity of HFOs for indicating EZ, endeavors have been made to distinguish events of interest. For example, Fabrice Wendling et al. used a similar two-stage approach to detect events of interest and identify FRs based on parameters extracted from Fourier transform or wavelet transform (Birot et al., 2013). Taking advantage of multiple handcrafted features such as power band ratio, spectral centroid, and entropy, Su et al. successfully divided HFOs candidates into several clusters based on unsupervised clustering algorithms, which increased the accuracy for pathological HFOs detection (Liu et al., 2018). In this study, we aimed at improving this situation using four well trained CNN classifiers after initial detection. To be specific, in the testing dataset from real patients, we termed the detected events from initial detector as cHFOs, which indicated that they cannot be directly deemed as HFOs without further artifacts rejection. As can be seen from Table 2, there were 13.80% false HFOs and they could make a big difference in the final results. The trained classifiers successfully improved the localization value of HFOs for SOZ by rejecting those artifacts. The SOZ localization value was further improved after the FR classifier was applied, suggested by the increased AUC values.

Clinically, physicians tend to use high-pass filtering of EEG signals to suppress background activity and highlight some oscillations in the frequency band of interest. However, both HFOs and sharp transients may be represented as in Amiri et al. (2016); in this scenario, it is often helpful to overview the broadband signal or the time-frequency scalogram, which manifests the full spectral characteristics of the HFO event, for categorization tasks. Inspired by current clinical workflows and different from unsupervised clustering, we chose the CNN model as our classifier, reflecting the idea from data to decisions. The development of machine learning in recent years has heavily emphasized and benefited from CNN, which was originally designed to handle object classification tasks. Across multiple layers, these networks extract features from low levels to higher levels, often described as end-to-end and inspired by the brain recognition process (Di Carlo and Cox, 2007). Among different CNN architectures, ResNet101 mitigates the problem of vanishing gradient resulting from improper hyperparameter tuning and the increased stacked layers by skip connections, and it is also actively chosen for computer vision tasks for its ability to generalize well to different datasets and problems (Wu et al., 2019). In contrast to handcrafted feature-based clustering, a deep learning neural network can automatically extract features and perform classification tasks. The key of this classification algorithm was to gather enough representative events as training materials so that the classifier can be trained toward good generalization ability. Because the imbalance distribution of different event types was shown in the results, it was more feasible to train four binary CNN classifiers rather than one multiclass classifier. Using transfer learning, it was possible to train a more generalized deep neural network for classification with limited samples.

The issue with the TF scalogram is that the low-frequency component may make HFOs less visible given specific settings because of the 1/f spectrum law. Researchers have designed various strategies, such as autoregressive integrated moving average, Teager-Kaiser operator energy and H_0_ *z*-score (Roehri et al., 2016) to flatten the spectrum. Here, we arbitrarily performed 8 Hz high-pass filtering of the raw trace and then log-transformed the raw energy. The spectrum was whitened, while most low-frequency components were preserved under such settings. In scalograms, true HFOs are visible as isolated “blobs” in the time-frequency plane, while the pure spike and the transient sharp artifact produce a single elongated shape with no visible band-limited blobs (Benar et al., 2010). During the manual labeling period, it was sometimes challenging to discern HFOs when the spikes co-occurred. We tended to check the raw and high-pass filtered trace as supplementary proof for visual classification. Based on the combined manually labeled and simulated dataset, the training and validation results of the four ResNet101 classifiers showed robust accuracies above 95% across different split ratios. The four successfully trained classifiers laid solid foundation for improving the localization value of HFOs.

After the classifiers were trained properly, we sought to further validate the classification performance in 20 real patients against 3 assumptions, which could be safely drawn from previous studies: (1) the EZ localization capability could be enhanced by removing artifacts and false HFOs (Benar et al., 2010); (2) HFOs cooccurring with spikes had higher localization value than those without (Wang et al., 2013; Weiss et al., 2016; Wang et al., 2017), and (3) FRs were more closely related to the EZ (Engel et al., 2009; Gonzalez Otarula et al., 2019). By labeling cHFOs, our detector successfully verified the assumptions concluded from previous studies. In addition, we demonstrated that the AUC values of qHFOs and qHFOs with FR clustered near 1. In the qHFOs with FR group, 19 out of 20 AUC values were above 0.8, and 15 were above 0.9. The results suggested the excellent ability to predict SOZ

channels using the classified HFOs rate. Poor concordance was found in two TSC patients, reflecting the multifocal nature and a complex widespread epileptic network in patients with TSC (Okanishi et al., 2014).

Aside from artifact removal, it was also important to separate pathological HFOs and physiological HFOs. Previous studies suggest that they could be distinguished based on their frequency band, their co-occurrence with interictal epileptiform discharges (Wang et al., 2013; Jacobs et al., 2016), their stereotyped morphology patterns and their spatial distribution (Liu et al., 2018). Therefore, successful classification of HFOs with FR or co-occurring with other interictal epileptiform discharges such as spike may help improve the specificity of HFOs in delineating EZ, which could be achieved through our algorithm.

Detection and classification algorithms tend to be optimized for recording a specific group with limited diversity in epilepsy syndromes, which is true for this automatic pipeline. Validation on a larger cohort from a multicenter is needed to better evaluate the prediction performance of this algorithm. Furthermore, because of the sophisticated design, this algorithm is computationally expensive compared with other detectors.

In this paper, we proposed an integrated pipeline for automatic detection, classification and imaging of HFOs with SEEG. Our initial detector demonstrated robust detection results on a comprehensive simulated dataset. The CNN-based classifiers achieved satisfactory accuracy, and their generalization ability was also validated in an extra real patient cohort. Thus, the proposed detection method dramatically decreased the workload in assessing the presence of HFOs in SEEG while providing straightforward interpretable results for surgical planning.

## Data Availability Statement

The datasets generated for this study are available on request to the corresponding author. Codes of the algorithms described in this paper, including the trained neural network, are open-source and openly available (https://github.com/zhaobaotian/HFO_AI_Detector_Open).

## Ethics Statement

The studies involving human participants were reviewed and approved by the Ethics Committee of Beijing Tiantan Hospital. Written informed consent to participate in this study was provided by the participants or their legal guardian/next of kin.

## Author Contributions

JZ and BZ conceived and planned the study. JZ and KZ performed the surgery resection. BZ performed the statistical analysis and drafted the manuscript. BZ, WH, and CZ worked out almost all the technical details. XW, YW, and CL collected the data. JM suggested the neural network architecture. XY and XS interpretation of the SEEG data. LS and YM discussed the results and revised the manuscript. All authors provided critical feedback and helped shape the research, analysis and the manuscript.

## Conflict of Interest

The authors declare that the research was conducted in the absence of any commercial or financial relationships that could be construed as a potential conflict of interest.

## Footnotes

1 https://www.fil.ion.ucl.ac.uk/spm/

2 https://github.com/zhaobaotian/HFO_AI_Detector_Open

## References

[B1] Aanestad E. Gilhus N. E. Brogger J. (2020). Interictal epileptiform discharges vary across age groups. *Clin. Neurophysiol.* 131 25–33. 10.1016/j.clinph.2019.09.017 31751836

[B2] Akiyama T. McCoy B. Go C. Y. Ochi A. Elliott I. M. Akiyama M. (2011). Focal resection of fast ripples on extraoperative intracranial EEG improves seizure outcome in pediatric epilepsy. *Epilepsia* 52 1802–1811. 10.1111/j.1528-1167.2011.03199.x 21801168

[B3] Alom M. Z. Taha T. M. Yakopcic C. Westberg S. Sidike P. Nasrin M. S. (2018). The history began from alexnet: a comprehensive survey on deep learning approaches. *arXiv* [Preprint]. arXiv:1803.01164.

[B4] Amiri M. Lina J. M. Pizzo F. Gotman J. (2016). High Frequency oscillations and spikes: separating real HFOs from false oscillations. *Clin. Neurophysiol.* 127 187–196. 10.1016/j.clinph.2015.04.290 26100149

[B5] Benar C. G. Chauviere L. Bartolomei F. Wendling F. (2010). Pitfalls of high-pass filtering for detecting epileptic oscillations: a technical note on “false” ripples. *Clin. Neurophysiol.* 121 301–310. 10.1016/j.clinph.2009.10.019 19955019

[B6] Birot G. Kachenoura A. Albera L. Bénar C. Wendling F. (2013). Automatic detection of fast ripples. *J. Neurosci. Methods* 213 236–249. 10.1016/j.jneumeth.2012.12.013 23261773

[B7] Bragin A. Engel J. Jr. Wilson C. L. Fried I. Mathern G. W. (1999). Hippocampal and entorhinal cortex high-frequency oscillations (100–500 Hz) in human epileptic brain and in kainic acid–treated rats with chronic seizures. *Epilepsia* 40 127–137. 10.1111/j.1528-1157.1999.tb02065.x 9952257

[B8] Bragin A. Mody I. Wilson C. L. Engel J. (2002). Local generation of fast ripples in epileptic brain. *J. Neurosci.* 22 2012–2021. 10.1523/jneurosci.22-05-02012.2002 11880532 PMC6758883

[B9] Burnos S. Hilfiker P. Sürücü O. Scholkmann F. Krayenbühl N. Grunwald T. (2014). Human intracranial high frequency oscillations (HFOs) detected by automatic time-frequency analysis. *PLoS One* 9:e94381. 10.1371/journal.pone.0094381 24722663 PMC3983146

[B10] Chaibi S. Sakka Z. Lajnef T. Samet M. Kachouri A. (2013). Automated detection and classification of high frequency oscillations (HFOs) in human intracereberal EEG. *Biomed. Signal Process. Control* 8 927–934. 10.1016/j.bspc.2013.08.009 28113293

[B11] Cimbalnik J. Brinkmann B. Kremen V. Jurak P. Berry B. Gompel J. V. (2018). Physiological and pathological high frequency oscillations in focal epilepsy. *Ann. Clin. Transl. Neurol.* 5 1062–1076. 10.1002/acn3.618 30250863 PMC6144446

[B12] Crepon B. Navarro V. Hasboun D. Clemenceau S. Martinerie J. Baulac M. (2010). Mapping interictal oscillations greater than 200 Hz recorded with intracranial macroelectrodes in human epilepsy. *Brain* 133(Pt 1) 33–45. 10.1093/brain/awp277 19920064

[B13] David O. Blauwblomme T. Job A. S. Chabardes S. Hoffmann D. Minotti L. (2011). Imaging the seizure onset zone with stereo-electroencephalography. *Brain* 134(Pt 10) 2898–2911. 10.1093/brain/awr238 21975587

[B14] Devinsky O. (1999). Patients with refractory seizures. *N. Engl. J. Med.* 340 1565–1570. 10.1056/NEJM199905203402008 10332020

[B15] Di Carlo J. J. Cox D. D. (2007). Untangling invariant object recognition. *Trends Cogn. Sci.* 11 333–341. 10.1016/j.tics.2007.06.010 17631409

[B16] Engel J. Jr. Bragin A. Staba R. Mody I. (2009). High-frequency oscillations: what is normal and what is not? *Epilepsia* 50 598–604. 10.1111/j.1528-1167.2008.01917.x 19055491

[B17] Frauscher B. Bartolomei F. Kobayashi K. Cimbalnik J. van ’t Klooster M. A. Rampp S. (2017). High-frequency oscillations: the state of clinical research. *Epilepsia* 58 1316–1329. 10.1111/epi.13829 28666056 PMC5806699

[B18] Gardner A. B. Worrell G. A. Marsh E. Dlugos D. Litt B. (2007). Human and automated detection of high-frequency oscillations in clinical intracranial EEG recordings. *Clin. Neurophysiol.* 118 1134–1143. 10.1016/j.clinph.2006.12.019 17382583 PMC2020804

[B19] Gliske S. V. Irwin Z. T. Davis K. A. Sahaya K. Chestek C. Stacey W. C. (2016). Universal automated high frequency oscillation detector for real-time, long term EEG. *Clin. Neurophysiol.* 127 1057–1066. 10.1016/j.clinph.2015.07.016 26238856 PMC4723299

[B20] Gonzalez Otarula K. A. von Ellenrieder N. Cuello-Oderiz C. Dubeau F. Gotman J. (2019). High-frequency oscillation networks and surgical outcome in adult focal epilepsy. *Ann. Neurol.* 85 485–494. 10.1002/ana.25442 30786048

[B21] He K. Zhang X. Ren S. Sun J. (2016). “Deep residual learning for image recognition,” in *Proceedings of the IEEE Conference on Computer Vision and Pattern Recognition*, (Piscataway, NJ: IEEE), 770–778.

[B22] Holler Y. Kutil R. Klaffenbock L. Thomschewski A. Holler P. M. Bathke A. C. (2015). High-frequency oscillations in epilepsy and surgical outcome. A meta-analysis. *Front. Hum. Neurosci.* 9:574. 10.3389/fnhum.2015.00574 26539097 PMC4611152

[B23] Iglesias J. E. Liu C.-Y. Thompson P. M. Tu Z. (2011). Robust brain extraction across datasets and comparison with publicly available methods. *IEEE Transact. Med. Imag.* 30 1617–1634. 10.1109/tmi.2011.2138152 21880566

[B24] Jacobs J. LeVan P. Chander R. Hall J. Dubeau F. Gotman J. (2008). Interictal high-frequency oscillations (80-500 Hz) are an indicator of seizure onset areas independent of spikes in the human epileptic brain. *Epilepsia* 49 1893–1907. 10.1111/j.1528-1167.2008.01656.x 18479382 PMC3792077

[B25] Jacobs J. Staba R. Asano E. Otsubo H. Wu J. Y. Zijlmans M. (2012). High-frequency oscillations (HFOs) in clinical epilepsy. *Prog. Neurobiol.* 98 302–315. 10.1016/j.pneurobio.2012.03.001 22480752 PMC3674884

[B26] Jacobs J. Vogt C. LeVan P. Zelmann R. Gotman J. Kobayashi K. (2016). The identification of distinct high-frequency oscillations during spikes delineates the seizure onset zone better than high-frequency spectral power changes. *Clin. Neurophysiol.* 127 129–142. 10.1016/j.clinph.2015.04.053 25998203

[B27] Jobst B. C. Cascino G. D. (2015). Resective epilepsy surgery for drug-resistant focal epilepsy: a review. *JAMA* 313 285–293. 10.1001/jama.2014.17426 25602999

[B28] Khadjevand F. Cimbalnik J. Worrell G. A. (2017). Progress and remaining challenges in the application of high frequency oscillations as biomarkers of epileptic brain. *Curr. Opin. Biomed. Eng.* 4 87–96. 10.1016/j.cobme.2017.09.006 29532041 PMC5844503

[B29] Kovach C. K. Tsuchiya N. Kawasaki H. Oya H. Howard M. A. III Adolphs R. (2011). Manifestation of ocular-muscle EMG contamination in human intracranial recordings. *Neuroimage* 54 213–233. 10.1016/j.neuroimage.2010.08.002 20696256 PMC2975438

[B30] Krizhevsky A. Sutskever I. Hinton G. E. (2012). Imagenet classification with deep convolutional neural networks. *Adv. Neural Inform. Process. Syst.* 25 1097–1105.

[B31] Liu S. Gurses C. Sha Z. Quach M. M. Sencer A. Bebek N. (2018). Stereotyped high-frequency oscillations discriminate seizure onset zones and critical functional cortex in focal epilepsy. *Brain* 141 713–730. 10.1093/brain/awx374 29394328 PMC6715109

[B32] Liu S. Sha Z. Sencer A. Aydoseli A. Bebek N. Abosch A. (2016a). Exploring the time-frequency content of high frequency oscillations for automated identification of seizure onset zone in epilepsy. *J. Neural Eng.* 13:026026. 10.1088/1741-2560/13/2/026026 26924828

[B33] Liu S. Sha Z. Sencer A. Aydoseli A. Bebek N. Abosch A. (2016b). Exploring the time–frequency content of high frequency oscillations for automated identification of seizure onset zone in epilepsy. *J. Neural Eng.* 13:026026. 10.1088/1741-2560/13/2/026026 26924828

[B34] Lopez-Cuevas A. Castillo-Toledo B. Medina-Ceja L. Ventura-Mejia C. Pardo-Pena K. (2013). An algorithm for on-line detection of high frequency oscillations related to epilepsy. *Comput. Methods Programs Biomed.* 110 354–360. 10.1016/j.cmpb.2013.01.014 23522965

[B35] Nagahama Y. Schmitt A. J. Nakagawa D. Vesole A. S. Kamm J. Kovach C. K. (2018). Intracranial EEG for seizure focus localization: evolving techniques, outcomes, complications, and utility of combining surface and depth electrodes. *J. Neurosurg.* 8 1–13. 10.3171/2018.1.JNS171808 29799342

[B36] Navarrete M. Alvarado-Rojas C. Le Van, Quyen M. Valderrama M. (2016). RIPPLELAB: a comprehensive application for the detection, analysis and classification of high frequency oscillations in electroencephalographic signals. *PLoS One* 11:e0158276. 10.1371/journal.pone.0158276 27341033 PMC4920418

[B37] Okanishi T. Akiyama T. Tanaka S. Mayo E. Mitsutake A. Boelman C. (2014). Interictal high frequency oscillations correlating with seizure outcome in patients with widespread epileptic networks in tuberous sclerosis complex. *Epilepsia* 55 1602–1610. 10.1111/epi.12761 25196064

[B38] Pantazis D. Weber D. L. Dale C. L. Nichols T. E. Simpson G. V. Leahy R. M. (2005). “Imaging of oscillatory behavior in event-related MEG studies,” in *Proceedings of SPIE-The International Society for Optical Engineering*, (Washington, DC: SPIE), 5674.

[B39] Roehri N. Lina J. M. Mosher J. C. Bartolomei F. Benar C. G. (2016). Time-frequency strategies for increasing high-frequency oscillation detectability in intracerebral EEG. *IEEE Trans. Biomed. Eng.* 63 2595–2606. 10.1109/TBME.2016.2556425 27875125 PMC5134253

[B40] Roehri N. Pizzo F. Bartolomei F. Wendling F. Benar C. G. (2017). What are the assets and weaknesses of HFO detectors? A benchmark framework based on realistic simulations. *PLoS One* 12:e0174702. 10.1371/journal.pone.0174702 28406919 PMC5390983

[B41] Rosenow F. Luders H. (2001). Presurgical evaluation of epilepsy. *Brain* 124(Pt 9) 1683–1700. 10.1093/brain/124.9.1683 11522572

[B42] Spring A. M. Pittman D. J. Aghakhani Y. Jirsch J. Pillay N. Bello-Espinosa L. E. (2018). Generalizability of high frequency oscillation evaluations in the ripple band. *Front. Neurol.* 9:510. 10.3389/fneur.2018.00510 30002645 PMC6031752

[B43] Staba R. J. Wilson C. L. Bragin A. Fried I. Engel J. Jr. (2002). Quantitative analysis of high-frequency oscillations (80-500 Hz) recorded in human epileptic hippocampus and entorhinal cortex. *J. Neurophysiol.* 88 1743–1752. 10.1152/jn.2002.88.4.1743 12364503

[B44] Thomschewski A. Hincapie A. S. Frauscher B. (2019). Localization of the epileptogenic zone using high frequency oscillations. *Front. Neurol.* 10:94. 10.3389/fneur.2019.00094 30804887 PMC6378911

[B45] Urrestarazu E. Chander R. Dubeau F. Gotman J. (2007). Interictal high-frequency oscillations (100-500 Hz) in the intracerebral EEG of epileptic patients. *Brain* 130(Pt 9) 2354–2366. 10.1093/brain/awm149 17626037

[B46] Vakharia V. N. Duncan J. S. Witt J. A. Elger C. E. Staba R. Engel J. Jr. (2018). Getting the best outcomes from epilepsy surgery. *Ann. Neurol.* 83 676–690. 10.1002/ana.25205 29534299 PMC5947666

[B47] van Klink N. E. C. Van’t Klooster M. A. Zelmann R. Leijten F. S. S. Ferrier C. H. Braun K. P. J. (2014). High frequency oscillations in intra-operative electrocorticography before and after epilepsy surgery. *Clin. Neurophysiol.* 125 2212–2219. 10.1016/j.clinph.2014.03.004 24704141

[B48] van ’t Klooster M. A. van Klink N. E. Leijten F. S. Zelmann R. Gebbink T. A. Gosselaar P. H. (2015). Residual fast ripples in the intraoperative corticogram predict epilepsy surgery outcome. *Neurology* 85 120–128. 10.1212/WNL.0000000000001727 26070338

[B49] Wang S. So N. K. Jin B. Wang I. Z. Bulacio J. C. Enatsu R. (2017). Interictal ripples nested in epileptiform discharge help to identify the epileptogenic zone in neocortical epilepsy. *Clin. Neurophysiol.* 128 945–951. 10.1016/j.clinph.2017.03.033 28412559

[B50] Wang S. Wang I. Z. Bulacio J. C. Mosher J. C. Gonzalez-Martinez J. Alexopoulos A. V. (2013). Ripple classification helps to localize the seizure-onset zone in neocortical epilepsy. *Epilepsia* 54 370–376. 10.1111/j.1528-1167.2012.03721.x 23106394

[B51] Weiss S. A. Orosz I. Salamon N. Moy S. Wei L. Van’t Klooster M. A. (2016). Ripples on spikes show increased phase-amplitude coupling in mesial temporal lobe epilepsy seizure-onset zones. *Epilepsia* 57 1916–1930. 10.1111/epi.13572 27723936 PMC5118142

[B52] Worrell G. A. Parish L. Cranstoun S. D. Jonas R. Baltuch G. Litt B. (2004). High-frequency oscillations and seizure generation in neocortical epilepsy. *Brain* 127(Pt 7) 1496–1506. 10.1093/brain/awh149 15155522

[B53] Wu J. Y. Sankar R. Lerner J. T. Matsumoto J. H. Vinters H. V. Mathern G. W. (2010). Removing interictal fast ripples on electrocorticography linked with seizure freedom in children. *Neurology* 75 1686–1694. 10.1212/WNL.0b013e3181fc27d0 20926787 PMC3033604

[B54] Wu Z. Shen C. Van Den Hengel A. (2019). Wider or deeper: revisiting the resnet model for visual recognition. *Pattern Recogn.* 90 119–133. 10.1016/j.patcog.2019.01.006

[B55] Zelmann R. Mari F. Jacobs J. Zijlmans M. Dubeau F. Gotman J. (2012). A comparison between detectors of high frequency oscillations. *Clin. Neurophysiol.* 123 106–116. 10.1016/j.clinph.2011.06.006 21763191 PMC3782488

[B56] Zijlmans M. Worrell G. A. Dumpelmann M. Stieglitz T. Barborica A. Heers M. (2017). How to record high-frequency oscillations in epilepsy: a practical guideline. *Epilepsia* 58 1305–1315. 10.1111/epi.13814 28622421

[B57] Zijlmans M. Zweiphenning W. van Klink N. (2019). Changing concepts in presurgical assessment for epilepsy surgery. *Nat. Rev. Neurol.* 15 594–606. 10.1038/s41582-019-0224-y 31341275

[B58] Zuo R. Wei J. Li X. Li C. Zhao C. Ren Z. (2019). Automated detection of high-frequency oscillations in epilepsy based on a convolutional neural network. *Front. Comput. Neurosci.* 13:6. 10.3389/fncom.2019.00006 30809142 PMC6379273

