# Supplementary material for: Integrated Automatic Detection, Classification and Imaging of High Frequency Oscillations With Stereoelectroencephalography
Source: Front Neurosci. 2020 Jun 4;14:546. doi: 10.3389/fnins.2020.00546 (PMC7287040; doi:10.3389/fnins.2020.00546)
Supplement: Supplementary file 1 [file Table_1.DOCX]

Supplementary Material

Supplementary table 1: Clinical information of 12 patients whose SEEG data were used for training neural network classifiers

| Patient | Gender  /age (years) | Epilepsy onset  (years) | MRI findings | Seizure type | SOZ | Surgery | Depth electrodes  (contacts × #) | Pathological results | Postoperative follow-up (month) | Outcome-Engel’s class |
| --- | --- | --- | --- | --- | --- | --- | --- | --- | --- | --- |
| 01 | F/29 | 8 | Encephalomalacia | PSz2G | R-P & p-I | TCR | 8 × 6 | FCD IIa | 7 | I |
| 02 | F/18 | 1 | L-HS | PSz | L-m-T | ATL | 16 × 4; 12 × 4 | HS | 12 | I |
| 03 | M/35 | 32 | Negative | PSz & PSz2G | R-m-T | ATL | 16 × 5; 12 × 6 | HS | 9 | I |
| 04 | F/17 | 12 | Negative | PSz & PSz2G | R-OFC | TCR | 16 × 3; 12 × 6; 8 × 1 | FCD Ib | 16 | II |
| 05 | M/19 | 3 | Negative | PSz2G | L-OFC | TCR | 16 × 3; 12 × 4 | Gliosis | 15 | I |
| 06 | M/13 | 9 | Negative | PSz | L-SFS | TCR | 16 × 1; 12 × 4; 8 × 2 | FCD IIb | 14 | I |
| 07 | M/33 | 11 | Negative | PSz & PSz2G | R-OFC & a-I | TCR | 16 × 5; 12 × 4 | FCD IIa | 12 | III |
| 08 | F/21 | 11 | Negative | PSz & PSz2G | L-m-F | Coagulation | 16 × 3; 12 × 7; 8 × 2 | N/A | 8 | I |
| 09 | F/23 | 13 | R-P lesion | PSz2G | R-p-I | TCR | 16 × 4; 12 × 6; 8 × 1 | FCD IIb | 8 | I |
| 10 | F/28 | 18 | R-HS | PSz | R-m-T | ATL | 16 × 4; 12 × 3; 8 × 2 | HS | 4 | I |
| 11 | F/7 | 6 | Negative | PSz2G | R-SFS | TCR | 12 × 3; 8 × 2 | FCD IIb | 6 | I |
| 12 | M22 | 11 | Negative | PSz & PSz2G | Wide spread | Not operated | 16 × 2; 12 × 5; 8 × 3 | N/A | N/A | N/A |

M: male; F: female; L: left; R: right; PSz: partial seizure; PSz2G: partial seizure secondarily generalized; SOZ: seizure onset zone; m: mesial; l: lateral; p: posterior; T: temporal; F: frontal; P: parietal; I: insular; OFC: orbital frontal cortex; SFS: superior frontal sulcus; TCR: tailored cortical resection; ATL: anterior neocortical temporal resection (plus selective amygdalo-hippocampectomy): FCD: focal cortical dysplasia; HS: hippocampal sclerosis; N/A: not applicable

Supplementary table 2: Clinical information of 20 patients for testing.

| Patient | Gender  /age (years) | Epilepsy onset  (years) | MRI findings | Seizure type | SOZ | SOZ channels# | surgery | Depth electrodes  (contacts × #) | Pathological results | Postoperative follow-up (month) |
| --- | --- | --- | --- | --- | --- | --- | --- | --- | --- | --- |
| 01 | M/10 | 9 | Subependymal nodules | PSz | L-m-F | 6 | TCR | 16 × 3; 12 × 6; 8 × 1 | TSC | 48 |
| 02 | M/14 | 4 | Negative | PSz2G | L-I | 4 | TCR | 16 × 1; 12 × 5; 10 × 4; 8 × 2 | FCD IIa | 48 |
| 03 | M/30 | 15 | Negative | PSz2G | R-I | 6 | TCR | 16 × 3; 12 × 1; 10 × 2; 8 × 1 | FCD IIa | 46 |
| 04 | M/21 | 5 | L-HS | PSz & PSz2G | L-m-T | 6 | ATL | 16 × 4; 12 × 5 | FCD IIIa | 46 |
| 05 | M/29 | 0.5 | Negative | PSz & PSz2G | R-m-T | 4 | ATL | 16 × 7; 12 × 2; 10 × 1 | HS | 42 |
| 06 | M/28 | 15 | Negative | PSz & PSz2G | L-SFS | 14 | TCR | 16 × 1; 12 × 2; 10 × 2; 8 × 1 | FCD IIa | 42 |
| 07 | M/25 | 4 | L-HS | PSz & PSz2G | L-m-T | 6 | ATL | 16 × 2; 12 × 2; 10 × 3; 8 × 3 | FCD IIIa | 41 |
| 08 | F/13 | 5 | Negative | PSz | L-I | 12 | TCR | 16 × 2; 12 × 2; 10 × 2 | FCD IIa | 39 |
| 09 | M/16 | 14.5 | Negative | PSz2G | R-OFC | 5 | TCR | 16 × 4; 12 × 3; 10 × 2 | FCD IIa | 38 |
| 10 | M/29 | 13 | L-HS | PSz | L-m-T | 7 | ATL | 16 × 3; 12 × 2; 10 × 1 | FCD IIIa | 38 |
| 11 | M/16 | 2 | Subependymal nodules | PSz | R-F nodule | 6 | TCR | 16 × 4; 12 × 4 | TSC | 37 |
| 12 | M/21 | 14 | Negative | PSz | R-OFC | 1 | TCR | 16 × 4; 12 × 3; 8 × 1 | FCD Ia | 37 |
| 13 | M/14 | 2 | Subependymal nodules | PSz & PSz2G | R-P nodule | 3 | TCR | 10 × 2; 8 × 6 | TSC | 37 |
| 14 | F/26 | 24.5 | Negative | PSz | R-m-T | 5 | ATL | 16 × 9; 12 × 1; 10 × 3; 8 × 2 | FCD IIIa | 35 |
| 15 | M/26 | 3 | L-hemisphere atrophy | PSz & PSz2G | L-m-T | 8 | ATL | 16 × 1; 12 × 6; 10 × 5 | FCD IIIa | 35 |
| 16 | F/23 | 12 | Negative | PSz | R-OFC & a-I | 6 | TCR | 16 × 3; 12 × 4; 10 × 1; 8 × 1 | FCD IIa | 34 |
| 17 | M/15 | 3 | Negative | PSz | L-OFC & a-I | 5 | TCR | 16 × 5; 12 × 4; 10 × 1 | Gliosis | 33 |
| ­18 | M/16 | 5 | L-F lesion | PSz & PSz2G | L-SFS | 7 | TCR | 16 × 1; 12 × 3; 10 × 1 | FCD IIb | 32 |
| 19 | M/25 | 14 | Negative | PSz & PSz2G | R-m-T & l-T | 9 | ATL | 16 × 4; 12 × 3; 10 × 2; 8 × 1 | FCD Ib | 30 |
| 20 | F/14 | 5 | Negative | PSz | R-p-l-F | 7 | TCR | 16 × 2; 12 × 2; 10 × 1; 8 × 2 | FCD IIb | 26 |

M: male; F: female; L: left; R: right; PSz: partial seizure; PSz2G: partial seizure secondarily generalized; SOZ: seizure onset zone; m: mesial; l: lateral; a: anterior; p: posterior; T: temporal; F: frontal; P: parietal; I: insular; OFC: orbital frontal cortex; SFS: superior frontal sulcus; TCR: tailored cortical resection; ATL: anterior neocortical temporal resection (plus selective amygdalo-hippocampectomy): FCD: focal cortical dysplasia; HS: hippocampal sclerosis; TSC: tuberous sclerosis complex.
